# SARS-CoV-2 seropositivity in African women living with HIV and their infants

**DOI:** 10.1186/s12879-024-09591-8

**Published:** 2024-07-11

**Authors:** Taguma A. Matubu, Nonhlanhla Yende-Zuma, Sean S. Brummel, Lynda Stranix-Chibanda, Lillian Wambuzi Ogwang, Sufia Dadabhai, Patience Atuhaire, Felluna Chauwa, Luis Gadama, Reinaldo E. Fernandez, Jim Aizire, JBrooks Jackson, Aaron A. R. Tobian, Taha E. Taha, Mary Glenn Fowler

**Affiliations:** 1 University of Zimbabwe Clinical Trials Research Centre, Harare, Zimbabwe; 2 Biostatistics Research Unit, South African Medical Research Council, Durban, South Africa; 3 Centre for the AIDS Programme of Research in South Africa (CAPRISA), Nelson R Mandela School of Medicine, University of KwaZulu-Natal, Durban, South Africa; 4 Harvard T.H. Chan School of Public Health, Boston, MA USA; 5 Faculty of Medicine and Health Sciences, Child and Adolescent Health Unit, University of Zimbabwe, Harare, Zimbabwe; 6 Makerere University-Johns Hopkins University (MU-JHU) Research Collaboration, Kampala, Uganda; 7 Johns Hopkins Bloomberg School of Public Health, Blantyre, Malawi; 8 Kamuzu University of Health Sciences-Johns Hopkins Research Project, Blantyre, Malawi; 9 University of Iowa, Iowa City, Iowa USA; 10 Johns Hopkins University School of Medicine, Baltimore, MD USA

**Keywords:** SARS-CoV-2, Seropositivity, WLHIV, Children

## Abstract

**Background:**

SARS-CoV-2 seropositivity data in women living with HIV (WLHIV), their infants and associated factors in this subpopulation remain limited. We retrospectively measured SARS-CoV-2 seropositivity from 07/2020-11/2021 among WLHIV and their children in the PROMOTE observational cohort in Uganda, Malawi, and Zimbabwe prior to widespread SARS-CoV-2 vaccination in those countries.

**Methods:**

Plasma stored during 3 waves of the COVID-19 pandemic in East/Southern Africa were tested for SARS-CoV-2 specific IgG antibodies (Ab) using serological assays that detect adaptive immune responses to SARS-CoV-2 spike protein. (*EUROIMMUN, Mountain Lakes, New Jersey and Roche Diagnostics, Indianapolis, IN)*. Modified-Poisson regression models were used to calculate prevalence rate ratios (PRR) and 95% confidence intervals (CI) to identify sociodemographic and clinical risk factors.

**Results:**

PROMOTE samples from 918 mothers and 1237 children were analysed. Overall, maternal SARS-CoV-2 seropositivity was 60.1% (95% CI: 56.9 -63.3) and 41.5% (95%CI: 38.8 – 44.2) for children. Non-breastfeeding mothers had a 31% higher risk of SARS-CoV-2 seropositivity compared to breastfeeding mothers (aPRR=1.31, 95%CI: 1.08-1.59). WLHIV with undetectable viral load had a 10% increased risk of SARS-CoV-2 seropositivity (aPRR=1.10, 95%CI: 0.89-1.37). Moreover, those who were normotensive had 12% increased risk SARS-CoV-2 seropositivity (aPRR= 1.12, 95% CI: 0.68-1.85) compared to women with hypertension. Children between 2 and 5 years had a 19% reduced risk of SARS-CoV-2 seropositivity (aPRR=0.81, 95%CI: 0.64-1.02) when compared to younger children. Mother/infant SARS-CoV-2 serostatuses were discordant in 346/802 (43.1%) families tested: mothers+/children- in 72.3%; mothers-/children+ in 26.3%; child+/sibling+ concordance was 34.6%.

**Conclusions:**

These SARS-CoV-2 seropositivity data indicate that by late 2021, about 60% of mothers and about 40% of children in a cohort of HIV-affected families in eastern/southern Africa had been infected with SARS-CoV-2. More mothers than their infants tested SARS-CoV-2+, likely due to a greater external exposure for mothers linked to daily routines/employment, and school closures. Breastfeeding was protective for mothers, likely because of higher likelihood of staying home with young children, and thus less exposure. Discordant results between children within the same families underscores the need to further understand transmission dynamics within households.

**Supplementary Information:**

The online version contains supplementary material available at 10.1186/s12879-024-09591-8.

## Background

The World Health Organization (WHO) declared the SARS-CoV-2 outbreak a global pandemic on March 11, 2020, following the emergence of a cluster of pneumonia cases in Wuhan, China in December 2019. The disease was later named COVID-19, whose aetiological agent was identified as a novel coronavirus (SARS-CoV-2) [1]. By May 2023, an estimated 765 million people had been infected by SARS-CoV-2 globally, with 8 million deaths recorded, a combined 174 000 of these in 47 African countries [2]. COVID-19 statistics in resource-limited settings is largely collated from national testing programs driven by clinical symptoms and thus may underestimate true spread of the disease. Consequently, there is a paucity of SARS-CoV-2 seropositivity data in the African region and most importantly among people living with HIV (PLWH) who may be at a higher risk of COVID-19 complications/mortality due to underlying compromised immunity.

Current data do not suggest higher SARS-CoV-2 infection rates among PLWH [3–6] which corroborates findings from an earlier study of blood donor samples in Uganda which reported significantly lower seropositivity to S antibody among HIV-seropositive individuals. Furthermore, some studies assessing COVID-19 severity among PLWH showed no clear evidence of poorer outcomes [7]. However, emerging data is reporting higher risk of morbidity [4] and mortality [8–11] among some HIV-SARS-CoV-2 co-infected patients, particularly those with low CD4 cell counts and unsuppressed HIV viraemia [12, 13]. Some studies have also postulated that use of some antiretroviral drugs may offer protection against severe outcomes among HIV/SARSCoV-2 co-infected patients [3, 14, 15]. In resource-limited settings with high background endemic infections, PLWH may be at greater risk of severe disease given the high prevalence of TB and malaria that may exacerbate both HIV and COVID-19 co-morbidities [16].

COVID-19 vulnerability has been linked to social and structural determinants like poverty, population density, and lack of access to health services, generating a syndemia of social and health related challenges [17]. In this regard, PLWH share many structural factors for heightened SARS-CoV-2 acquisition and severity of the co-infection. PLWH have been shown to have a higher predisposition to respiratory infections due to compromised immunity when compared to the general population [18].

Data on population-level exposure and immunity to SARS-CoV-2 are fundamental in understanding transmission dynamics, assess population level susceptibility, and inform public health responses. Notably, estimating the proportion of the population that has been infected, particularly in resource-constrained settings, is compounded by asymptomatic or subclinical infections, inadequate testing capacity, and challenges in collecting routine surveillance data [19]. Seropositivity surveys can overcome these issues by identifying antibody responses that reflect prior SARS-CoV-2 exposure.

The PEPFAR-PROMOTE study was a longitudinal five-year follow-up roll over protocol that enrolled mothers living with HIV and their HIV exposed children previously enrolled in the IMPAACT PROMISE perinatal clinical trial from eight high enrolling African sites in the PROMISE trial (NCT01061151/NCT01253538, 2010/02/02). The PROMOTE study which began in 2016 enrolled 3771 mothers and children across the eight sites in Malawi, Uganda, Zimbabwe, and South Africa. The aims of the PROMOTE study were to follow-up on maternal and child health and survival over five years of PROMOTE follow up; assess factors that affect their morbidity and mortality; maternal adherence over time to lifetime ART, longer term complications related to the HIV disease progression, ART, and co-infections among mothers living with HIV; as well as health, survival, growth, and developmental outcomes among their HIV exposed but uninfected children [20]. In the current study, we retrospectively conducted a SARS-CoV-2 seropositivity descriptive study to determine sociodemographic and clinical risk factors, maternal and pregnancy outcomes among Women Living with HIV (WLHIV) and their infants enrolled in the PEPFAR-PROMOTE observational cohort in Uganda, Malawi, and Zimbabwe. For the seropositivity surveillance analyses, data from 918 mothers and 1237 children from these sites (Malawi (Blantyre), Uganda and Zimbabwe) were included.

## Methods

### Study design and participants

We conducted a retrospective, cross-sectional survey to estimate SARS-CoV-2 antibody prevalence within the PEPFAR-PROMOTE observational cohort study in the midst of the COVID-19 pandemic in Eastern and Southern Africa; and before widespread COVID-19 vaccine immunisations in Malawi, Uganda, and Zimbabwe were available. The most recent or study exit visit sample was tested for each participant. All PROMOTE study participants exited the study between July 2020 and November 2021. All sites are in urban/semi-urban settings; and all women enrolled in PROMOTE were on lifelong antiretroviral therapy (ART) that conform with each country's national guidelines as the prevailing standard of care and are not provided by the study [20].

### Study procedures

In the PROMOTE study, maternal follow-up evaluations were conducted every six months to collect data on medical history, ART use and adherence, physical examination, laboratory test results, and new events occurring between visits. The laboratory evaluations included viral load (HIV RNA PCR) testing every six months and complete blood count (CBC), CD4+ cell count, and serum chemistry (ALT, creatinine, and creatinine clearance) every 12 months. Blood was collected at scheduled six-monthly visits and processed plasma was stored at -80°C at the participating laboratories. In this sub-study, only samples collected at the latest visit for each participant were tested for SARS-CoV-2 antibodies and included in the analysis.

### Laboratory methods

Available stored plasma samples from 918 mothers and 1237 children enrolled during PEPFAR-PROMOTE study collected between July 2020 and November 2021 were included in this analysis. For Malawi and Zimbabwe cohorts, plasma was tested using the EUROIMMUN Anti-SARS-CoV-2 ELISA (IgG) antibody assay following kit-specific instructions (*EUROIMMUN, Mountain Lakes, New Jersey*). The EUROIMMUN Anti-SARS-CoV-2 ELISA (IgG) is an enzyme-linked immunosorbent assay intended for the qualitative detection of IgG class antibodies to SARS-CoV-2 to the Spike-1 protein.

For the Uganda cohort, plasma samples were tested with the Roche SARS-CoV-2 IgG antibody test kits, according to the package insert using the anti-SARS-CoV-2 immunoassay (Roche Diagnostics, Indianapolis, IN) for the qualitative detection of antibodies to SARS-CoV-2. The assay uses a recombinant protein representing the nucleocapsid (N) antigen for the determination of antibodies against SARS-CoV-2. The test is used as an aid in identifying individuals with an adaptive immune response to SARS-CoV-2, indicating recent or prior infection. The samples were also tested according to the package insert using the anti-SARS-CoV-2 S electrochemiluminescence immunoassay (Roche Diagnostics) for qualitative and semi-quantitative detection of antibodies to SARS-CoV-2 spike (S) protein receptor binding domain (RBD) in human serum and plasma.

### Statistical analysis

Maternal and infant overall SARS-CoV-2 antibody prevalence and 95% confidence intervals (CI) were calculated and reported. The confidence intervals were calculated using the score test method [21]. We utilised modified-Poisson regression models to calculate prevalence rate ratios (PRR) and 95% confidence intervals (CI) to identify marginal and conditional sociodemographic and clinical risk factors associated with SARS-CoV-2 seropositivity. Each univariable model included country. Each multivariable model included country and maternal risk factors including maternal age, employment status, marital status, level of education, availability of tap water at home, the distance between clinic and home, whether women have hypertension or not, whether they are breastfeeding or not, ART regimen and viral load. Children risk factors included country and age of the infant. SAS version 9.4 (*SAS Institute, Cary, NC*) was used for analyses.

### Ethical considerations

The PROMOTE study was approved by all relevant institutional review boards (IRBs) and women signed a written informed consent form to enrol and be followed-up with their children for five years and to provide study samples as part of their every six-month evaluation. Only samples from participants who provided consent for testing on stored samples were included in this analysis.

## Results

**Fig. 1:**
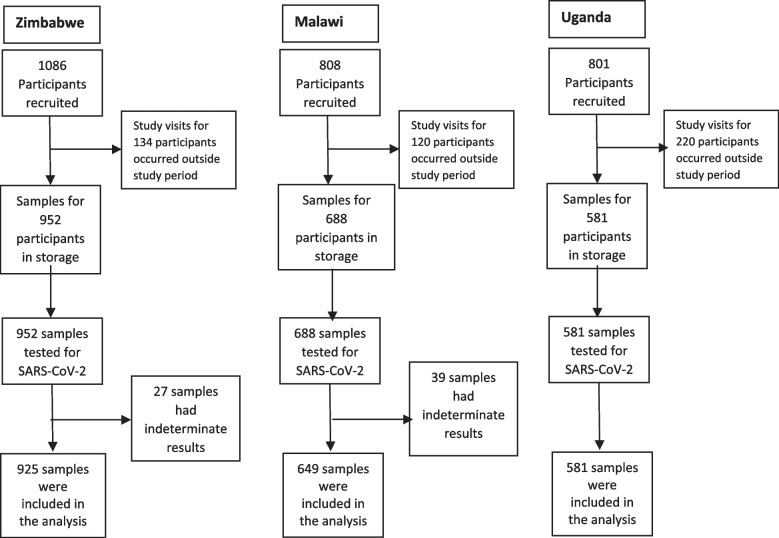
Flowchart for participants in seropositivity of SARS-CoV-2 antibodies study in three African countries between July 2020 and November 2021

The study analysed samples from a total of 2155 participants (918 mothers and 1237 children) from 3 countries (Zimbabwe – 925, Malawi – 649 and Uganda-581) collected between July 2020 and November 2021 (Fig 1). Out of a total of 1237 children included in the study, 19 (1.54%) were living with HIV (Zimbabwe – 7, Malawi – 6 and Uganda - 6) The baseline characteristics were comparable between participants testing positive (+) and negative (-) for SARS-CoV-2 Ab (Table 1)

**Table 1:** A & B: SARS-CoV-2 antibody seropositivity for mothers (A) and their children (B) across different characteristics and distribution of variables across the two groups

**A**	**Maternal Variable**	**Level**	**Positive (*N*=552)**	**Negative (*N*=366)**
	Country, n (%)	Uganda	131 (46.5%)	151 (53.5%)
		Malawi	160 (61.1%)	102 (38.9%)
		Zimbabwe	261 (69.8%)	113 (30.2%)
	Age (years), median (IQR) ^cd^		36 (33 - 40)	36 (32 - 39)
	Marital status, n (%)	No regular partner	19 (52.8%)	17 (47.2%)
		Primary regular partner	65 (54.2%)	55 (45.8%)
		Married	386 (62.6%)	231 (37.4%)
		Separated	35 (52.2%)	32 (47.8%)
		Divorced	15 (57.7%)	11 (42.3%)
		Widowed	32 (61.5%)	20 (38.5%)
	Highest level of education, n (%)	No schooling	11 (42.3%)	15 (57.7%)
		Primary-partially completed	101 (54.6%)	84 (45.4%)
		Primary-completed	54 (58.1%)	39 (41.9%)
		Secondary-partially completed	179 (59.7%)	121 (40.3%)
		Secondary completed	192 (69.3%)	85 (30.7%)
		College/University	15 (40.5%)	22 (59.5%)
	Employment status, n (%)	Formal employment	71 (55.5%)	57 (44.5%)
		Self-employment (Small business)	235 (60.6%)	153 (39.4%)
		Not employed/housewife	246 (61.2%)	156 (38.8%)
	No. people sleep in your room, median (IQR)^d^		3 (2 - 3)	3 (2 - 4)
	No. of children alive at baseline, median (IQR)*		3 (2 - 3)	3 (2 - 4)
	Currently breastfeeding, n (%) ^c^	Yes	55 (48.2%)	59 (51.8%)
		No	497 (61.8%)	307 (38.2%)
	BMI (kg/m^2^), median (IQR)^bcd^		27.0 (23.3 - 31.4)	26.1 (23 - 30.4)
	Tap water in the premises, n (%)	Yes	339 (62.7%)	202 (37.3%)
		No	213 (56.5%)	164 (43.5%)
	Travel time to the clinic, n (%)	Less than 30 minutes	156 (67.0%)	77 (33.0%)
		30-60 minutes	225 (61.6%)	140 (38.4%)
		1-2 hours	133 (56.1%)	104 (43.9%)
		Greater than 2 hours	38 (45.8%)	45 (54.2%)
	Viral load (copies/ml), n (%) ^ac^	Detectable	36 (56.3%)	28 (43.8%)
		Undetectable (incl <20:<40)	516 (60.4%)	338 (39.6%)
	ART regimen, n (%) ^c^	EFV/NVP based	70 (59.3%)	48 (40.7%)
		DTG based	446 (60.8%)	288 (39.2%)
		Lpv/r, ATV/r, RTV based	29 (51.8%)	27 (48.2%)
		Other	1 (100.0%)	0
		Stopped ART	6 (66.7%)	3 (33.3%)
	Hypertension, n (%) ^c^	No	545 (60.2%)	361 (39.8%)
		Yes	7 (58.3%)	5 (41.7%)
**B**	**Paediatric Variable**	**Level**	**Positive (*N*=513)**	**Negative (*N*=724)**
	Country, n (%)	Uganda	65 (21.7%)	234 (78.3%)
		Malawi	181 (46.8%)	206 (53.2%)
		Zimbabwe	267 (48.5%)	284 (51.5%)
	Age group, (years) n (%)^c^	<2	63 (42.9%)	84 (57.1%)
		2-5	106 (35.9%)	189 (64.1%)
		>5	344 (43.3%)	451 (56.7%)
	Sex, n (%)	Male	233 (38.3%)	375 (61.7%)
		Female	280 (44.5%)	349 (55.5%)
	Children living with HIV	Uganda	1 (16.7%)	5 (83.3%)
		Malawi	2 (33.3%)	4 (66.7%)
		Zimbabwe	2 (25.6%)	5 (71.4%)

^a^886 (96.5%) has VL done on the day of sample collection

^b^908 (98.9%) had BMI done on the day of sample collection

^c^Measurements done on or before sample collection

^d^Represents median for each group

**Fig. 2:**
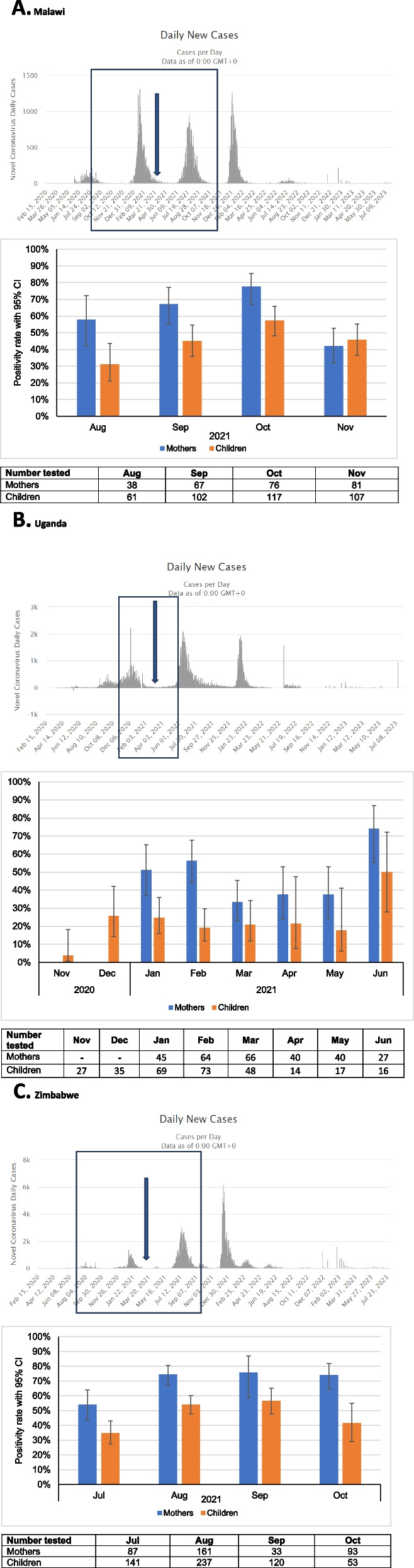
SARS-CoV-2 antibody seropositivity estimates over time for Malawi, Uganda, and Zimbabwe. **A** Shows SARS-CoV-2 antibody seropositivity rates over time for mothers and their children relative to COVID-19 trends over time in Malawi. Arrow depicts the period during which national COVID-19 vaccine program was launched. Rectangular boundary represents the study sampling period. **B** Shows SARS-CoV-2 antibody positivity rates over time for mothers and their Children relative to COVID-19 trends over time in Uganda. Arrow depicts the period during which national COVID-19 vaccine program was launched. Rectangular boundary represents the study sampling period. **C** Shows SARS-CoV-2 antibody positivity rates over time for mothers and their Children relative to COVID-19 trends over time in Zimbabwe. Arrow depicts the period during which national COVID-19 vaccine program was launched. Rectangular boundary represents the study sampling period

Overall, maternal SARS-CoV-2 seropositivity was 60.1% (95% CI: 56.9 -63.3) and 41.5% (95% CI: 38.8 – 44.2) for children. Country-specific SARS-CoV-2 seropositivity ranged between 46.5% to 69.8% among women and between 21.7% and 48.5% among children. Figure 2A-C shows the rates of SARS-CoV-2 antibody seropositivity over time and the periods during which COVID-19 vaccination was launched for each country.

Mothers who were not breastfeeding prior to sample collection were associated with a 31% (aPRR=1.31, 95%CI: 1.08-1.59) increased risk of SARS-CoV-2 seropositivity compared to those who were breastfeeding. Mothers with undetectable viral load had a 10% increased risk of SARS-CoV-2 seropositivity (aPRR=1.10, 95%CI: 0.89-1.37). Moreover, those who were not hypertensive had 12% increased risk SARS-CoV-2 seropositivity (aPRR= 1.12, 95% CI: 0.68-1.85) compared to those with hypertension (Table 2).

**Table 2:** Maternal factors associated with SARS-CoV-2 seropositivity

**Variable**	**PRR**^a^ **(95% CI)**		**aPRR(95% CI)**	***p*-value**
Age (5 years increase)	1.01 (0.96-1.06)	0.611	1.01 (0.95-1.08)	0.703
Primary school complete (ref: incomplete)	1.06 (0.91-1.23)	0.476	1.05 (0.90-1.22)	0.565
Married/regular partner (ref: not married)	1.05 (0.92-1.21)	0.447	1.05 (0.92-1.20)	0.495
Tap water at home (ref: No)	1.05 (0.94-1.17)	0.356	1.03 (0.92-1.15)	0.59
Travel <1 hour to clinic (ref: ≥1 hour)	1.06 (0.94-1.20)	0.341	1.03 (0.91-1.17)	0.634
Employment (ref: unemployed)				
Formal employment	0.99 (0.84-1.18)	0.929	0.98 (0.82-1.16)	0.796
Self employed	1.05 (0.94-1.17)	0.411	1.04 (0.93-1.17)	0.453
For every additional person sleeping in your room	1.02 (0.97-1.08)	0.41	1.03 (0.97-1.09)	0.293
For every additional child alive at baseline	1.00 (0.95-1.04)	0.821	0.98 (0.93-1.04)	0.592
Not breastfeeding (ref: Yes)	1.32 (1.09-1.60)	0.005	1.31 (1.08-1.59)	0.007
Undetectable viral load (ref: Detectable)	1.10 (0.89-1.36)	0.387	1.10 (0.89-1.37)	0.373
ART regimen (ref: EFV based regimen)				
DTG based regimen	1.03 (0.88-1.21)	0.723	1.02 (0.87-1.19)	0.843
PI based regimen	1.02 (0.76-1.36)	0.914	1.01 (0.75-1.37)	0.925
No Hypertension (ref: Hypertension)	1.13 (0.69-1.84)	0.63	1.12 (0.68-1.85)	0.648

*PRR* Prevalence rate ratio

Modified-Poisson regression models was used to calculate prevalence rate ratio and 95% CI

^a^each of the univariable models and a multivariable model were adjusted for the country variable

Children between 2 and 5 years had a 19% reduced risk of SARS-CoV-2 seropositivity (aPRR=0.81, 95%CI: 0.64-1.02) when compared to younger children. Mother/infant SARS-CoV-2 serostatuses were discordant in 346/802 (43.1%) families tested: mothers+/children- in 72.3%; mothers-/children+ in 26.3%; child+/sibling+ concordance was 34.6%.

## Discussion

Our data show that by November 2021, about 60% of mothers and about 40% of children in a cohort of HIV-affected families in eastern/southern Africa had been infected with SARS-CoV-2. The highest SARS-CoV-2 antibody seropositivity rates stood at 69.8% and 48.5% for WLHIV and children who were enrolled in the PROMOTE study in Zimbabwe respectively. In Malawi, we report 61.1% and 46.8% SARS-CoV-2 in WLHIV and their children at the end of October 2021. The seropositivity rates in our study are much higher than the 53% seropositivity estimate reported from a sample of blood donors which suggests higher rates of SARS-Cov-2 infection among WLHIV relative to the general population over this period [22]. For Uganda, our study shows much lower SARS-CoV-2 antibody seropositivity rates of 46.5% in WLHIV and 21.7% in children. The SARS-CoV-2 seropositivity rates in the Ugandan PROMOTE cohort were much lower than the 67.7% reported from another cohort during the same period [23]. The lower antibody seropositivity rates in Uganda may be attributable to a number of factors strict implementation of non-pharmaceutical Interventions such as hand washing, social distancing, wearing masks, prolonged school closures and partly due to the later emergence of COVID in that country. The Ugandan legislative framework enacted laws that resulted in implementation of broad public health measures restricting movement of vehicles, vessels, and aircraft,closure of international borders (except for cargo); closure of education facilities; closure of places of prayers; curfew, and mandatory wearing of facial masks [24]. Striking similarities in infection rates between Malawi and Zimbabwe for both mothers and children may be related to overlapping cultural and socio-behavioural norms between the two countries and similar timing of the pandemic emerging in both countries.

Our study was conducted when COVID-19 vaccination coverage was still extremely low in all 3 countries. The Zimbabwean Government rolled out COVID-19 vaccination in February 2021 with priority being given to health care workers. By the end of October 2021, only 10% of the Zimbabwean population had been vaccinated [25]. The PROMOTE study recruited participants mainly from informal dwellings and peri-urban areas who did not have ready access to COVID-19 vaccination until late 2021 and thus the vaccination rates are likely to be much lower in this group during the time that our study was conducted. COVID-19 vaccination program in Malawi was launched on 11 March 2021,however, the Government`s heavy reliance on COVAX for vaccine supply hugely constrained its rollout; and there were only small, sporadic shipments of AstraZeneca, Johnson & Johnson, and Pfizer vaccines. As a result, the COVID-19 vaccine coverage only reached 3% by November 2021. COVID-19 vaccination rates in Uganda were low with only 2% of the population being vaccinated at the end of August 2021 [25]. These data show that although COVID-19 vaccination started during a period that overlaps with our study, vaccination rates were extremely low and unlikely to affect the SARS-CoV-2 serostatus data generated. Additionally, for Uganda, testing was also done using recombinant protein representing the nucleocapsid (N) antigen which was able to confirm COVID-19 infection as opposed to seropositivity due only to spike protein related to receipt of vaccine.

Risk factor analysis in our study was meant to gain an understanding of whether preexisting conditions, medication for chronic illnesses and sociobehavioral changes may have modified risk of contracting COVID-19. In the African context, we reckon that breastfeeding women were likely to have stayed home with their breastfeeding children due to perceived risk to the infants, consequentially lessening exposure and rates of infection. WLHIV constantly need to visit health care facilities to replenish their medication, and this had potential to increase risk of infection in this sub population. We think understanding these dynamics is important to assess vulnerability of different groups in pandemic situations and thus any knowledge gleaned from current studies could be key in informing future public health approaches. Our results showed a comparable risk of SARS-CoV-2 antibody seropositivity among WLHIV who were on DTG and PI based regimens when compared to those on EFV. This finding is supported by findings from previously published studies which reported no differences in SARS-CoV-2 prevalence between PLWHIV on different ART regimens, suggesting ART does not offer differential protection against SARS-CoV-2 infection [26–28]. However, a study by Del Amo *et al* reported lower risk of infection and related hospitalisation in patients receiving TDF/FTC than those receiving other regimens suggesting the ART regimen has an impact on risk of SARS-CoV-2 infection [3].

Risk factors for post-acute sequelae of SARS-CoV-2 infection (PASC) have generated interest, with recent evidence showing that vaccination reduces but does not exclude the risk of PASC or “long COVID” [29]. Considering concerns about higher COVID-19 [30], increased frequency of autoimmune conditions [31], other medical comorbidities, and sub-optimal vaccine responses among PLWH, further research is urgently needed to ascertain longer-term COVID-19 outcomes among PLWH on different ART regimens.

The current study showed slightly higher SARS-CoV-2 prevalence in children below the age of two years and those above the age of five years compared to the age groups between two and five years. These results partly corroborate findings from a study by Huete-Pérez et al that reported higher SARS-CoV-2 rates in children below 5 years when compared to older counterparts in Nicaragua [32]. The high rates of infection in the age groups below two years and those above 5 years maybe be due to the children above 5 years attending school and interacting with peers outside the home during the pandemic. The mother/infant SARS-CoV-2 antibody results which show that more mothers were infected than their infants may explain the higher seropositivity for children below two years of age who spent a significant amount of time with their mothers for care. This also provides an insight into complex household transmission dynamics that require further investigation. One likely explanation for higher rates of seropositivity in mothers could be the greater need for them to undertake daily routines outside the home, such as going to work, against a background of school closures during the pandemic and increasing risk of infection through out of the home interactions. The strengths of our study include the relatively large sample size and robust demographic and clinical data generated during execution of the PEPFAR PROMOTE study. Furthermore, we used a testing methodology which has been rigorously validated on thousands of samples with high accuracy and precision.

Our study has some limitations. Most participants included in our analysis were on highly active antiretroviral therapy and suppressed HIV viral load for prolonged periods. The women in our study have been in research settings for a long time and have received excellent health services and health education about infection prevention. Therefore, these findings may not be generalizable to WLHIV in the general population who may not be as empowered or able to access regular health care services. Furthermore, our data are derived from three countries that experienced COVID-19 peaks at different times. Our study also employed two different validated SARS-CoV-2 antibody assays across the 3 countries, with Uganda using an assay that is different from the one used in Zimbabwe and Malawi. Variability in kit sensitivity may cause slight differences in positivity rates, making data comparison across countries less reliable. Our study did not perform additional testing to differentiate whether the detected antibodies in children below the age of 2 years were due to infection or passive transmission from the infected mothers. A recent study reported a significant correlation between SARS-CoV-2 IgG antibodies in maternal and cord blood, suggesting that indeed these antibodies can be transferred from mother to child [33].

## Conclusions

We found evidence of a high rate of SARS-CoV-2 infections in Southern and Eastern Africa following the second wave infection that occurred between July and November 2021. These SARS-CoV-2 serostatus data indicate that by late 2021, more than half of mothers and more than a third of children in a cohort of HIV-affected families in eastern/southern Africa had been infected with SARS-CoV-2. Breastfeeding women may have modified social behavioural routines that may modify their risk of contracting disease in a pandemic situation. The data also provides evidence that ART regimen does not alter risk of SARS-CoV-2 seropositivity further strengthening data that suggests that ART offers no protection against COVID-19.

### Supplementary Information

Supplementary Material 1. 

## Data Availability

De-identified participant data results will be shared upon reasonable request within one year of study publication upon submission of a data request form to the corresponding author (amatubu@uz-ctrc.org). All data sharing guidelines of the sponsors will be followed. Approval of the data request form (provided by the corresponding author) and signing of a data access agreement form will be required
