# Supplementary material for: SARS-CoV-2 seropositivity in African women living with HIV and their infants
Source: BMC Infect Dis. 2024 Jul 11;24:693. doi: 10.1186/s12879-024-09591-8 (PMC11241888; doi:10.1186/s12879-024-09591-8)
Supplement: Supplementary file 1 — Supplementary Material 1. [file 12879_2024_9591_MOESM1_ESM.docx]

**Supplemental Table 1: SARS-CoV-2 antibody results**

| **Test name** | **Group** | **Positive** | **Negative** | **Reactive** | **Non-reactive** | **Indeterminate** | **Total** |
| --- | --- | --- | --- | --- | --- | --- | --- |
| **Zimbabwe** antibody results (anti-S receptor binding domain) | Children | 267 (48.5%) | 284 (51.5%) | N/A | N/A | 15 (2.6%) | **566** |
|  | Mothers | 261 (69.8%) | 113 (30.2%) | N/A | N/A | 12 (3.0%) | **386** |
|  |  |  |  |  |  |  |  |
| **Malawi** antibody results (anti-S receptor binding domain) | Children | 181 (46.8%) | 206 (53.2%) | N/A | N/A | 20 (4.5%) | **407** |
|  | Mothers | 160 (61.1%) | 102 (38.9%) | N/A | N/A | 20 (6.5%) | **282** |
|  |  |  |  |  |  |  |  |
| **Uganda** antibody results (anti-S receptor binding domain) | Children | 65 (18.7%) | 282 (81.3%) | N/A | N/A | N/A | **347** |
|  | Mothers | 131 (46.5%) | 151 (53.5%) | N/A | N/A | N/A | **282** |
|  |  |  |  |  |  |  |  |
| **Uganda** Anti SARS CoV-2 nucleocapsid | Children | N/A | N/A | 60 (17.3%) | 285 (82.1%) | 2 (0.6%) | **347** |
|  | Mothers | N/A | N/A | 132 (42.7%) | 149 (57.0%) | 1 (0.3%) | **282** |
|  |  |  |  |  |  |  |  |

Table shows SARS-CoV-2 results by laboratory testing method and country
